# High-altitude deer mouse hypoxia-inducible factor-2α shows defective interaction with CREB-binding protein

**DOI:** 10.1016/j.jbc.2021.100461

**Published:** 2021-02-25

**Authors:** Daisheng Song, Abigail W. Bigham, Frank S. Lee

**Affiliations:** 1 Department of Pathology and Laboratory Medicine, Perelman School of Medicine, University of Pennsylvania, Philadelphia, Pennsylvania, USA; 2 Department of Anthropology, University of California, Los Angeles, California, USA

**Keywords:** evolution, factor inhibiting HIF, FIH, HIF, high-altitude adaptation, hypoxia, hypoxia-inducible factor, hydroxylase, transcription factor, CBP, CREB-binding protein, CTAD, C-terminal transactivation domain, FIH, factor inhibiting HIF, HIF, hypoxia-inducible factor, HIF-α, α subunit of the transcription factor HIF, ODD, oxygen-dependent degradation, PHD2, prolyl hydroxylase domain protein 2, VHL, von Hippel Lindau

## Abstract

Numerous mammalian species have adapted to the chronic hypoxia of high altitude. Recent genomic studies have identified evidence for natural selection of genes and associated genetic changes in these species. A major gap in our knowledge is an understanding of the functional significance, if any, of these changes. Deer mice (*Peromyscus maniculatus*) live at both low and high altitudes in North America, providing an opportunity to identify functionally important genetic changes. High-altitude deer mice show evidence of natural selection on the *Epas1* gene, which encodes for hypoxia-inducible factor-2α (Hif-2α), a central transcription factor of the hypoxia-inducible factor pathway. An SNP encoding for a T755M change in the Hif-2α protein is highly enriched in high-altitude deer mice, but its functional significance is unknown. Here, using coimmunoprecipitation and transcriptional activity assays, we show that the T755M mutation produces a defect in the interaction of Hif-2α with the transcriptional coactivator CREB-binding protein. This results in a loss of function because of decreased transcriptional activity. Intriguingly, the effect of this mutation depends on the amino acid context. Interchanges between methionine and threonine at the corresponding position in house mouse (*Mus musculus*) Hif-2α are without effects on CREB-binding protein binding. Furthermore, transfer of a set of deer mouse–specific Hif-2α amino acids to house mouse Hif-2α is sufficient to confer sensitivity of house mouse Hif-2α to the T755M substitution. These findings provide insight into high-altitude adaptation in deer mice and evolution at the *Epas1* locus.

The chronic hypoxia of high altitude presents a substantial challenge to metazoans residing in this environment. Recent studies have revealed evidence of genetic adaptation to high altitude in multiple mammalian species. These include humans who reside on the Tibetan plateau, the Andean Altiplano, and Ethiopian Simien Mountains (1), as well as Tibetan dogs, yaks, sheep, and horses (2, 3).

In many of these species, there is evidence for natural selection acting on genes of the hypoxia-inducible factor (HIF) pathway, the main transcriptional pathway by which cells respond to hypoxia (4, 5, 6). The HIF pathway relies on two oxygen-dependent enzymes that act on the α subunit of the transcription factor HIF (HIF-α). One is prolyl hydroxylase domain protein 2 (PHD2), which catalyzes prolyl hydroxylation in the oxygen-dependent degradation (ODD) domain of HIF-α (7, 8, 9). Prolyl hydroxylated HIF-α (of which there are two main paralogues, HIF-1α and HIF-2α) is recognized by the von Hippel Lindau (VHL) protein, which then targets HIF-α for degradation. Under hypoxia, prolyl hydroxylation is arrested, leading to the stabilization of HIF-α. HIF-α dimerizes with aryl hydrocarbon receptor nuclear translocator through their basic Helix Loop Helix-Period Arnt Sim domains to form a transcription factor complex.

The other critical oxygen-dependent enzyme in this pathway is factor inhibiting HIF (FIH), which catalyzes asparaginyl hydroxylation in the C-terminal transactivation domain (CTAD) of HIF-α (10, 11, 12). This hydroxylation blocks the interaction of the CTAD with CREB-binding protein (CBP), a transcriptional coactivator with lysine acetyltransferase activity. CBP is a component of the transcription preinitiation complex, and it acetylates lysines 27 and 18 on histone H3, marks characteristic of active chromatin (13). Under hypoxia, asparaginyl hydroxylation is arrested, leading to the binding of HIF-α to CBP and activation of the CTAD.

HIF activates hundreds of genes involved in cellular and systemic responses to hypoxia (14). HIF-1α, which is ubiquitously expressed, upregulates the genes of glycolysis and thereby promotes a shift from oxidative phosphorylation to anaerobic glycolysis. HIF-2α, with a more restricted expression that is tissue and cell type specific, plays a critical role in other aspects of the hypoxic response (15, 16, 17, 18).

North American deer mice (*Peromyscus maniculatus*) reside at both low and high altitudes, providing an opportunity to examine genes that might facilitate hypoxic adaption (19). One mechanism by which this occurs is through amino acid substitutions in the α and β chains of hemoglobin that increase binding affinity for oxygen (20). It has recently been reported that the *Epas1* gene, which encodes for Hif-2α, is under natural selection in high-altitude deer mice (21). The *Epas1* gene is the target of selection in multiple high-altitude species (2, 3). However, there is a dearth of knowledge regarding mechanisms by which high-altitude *Epas1* alleles might be adaptive. In deer mice, the high-altitude allele is correlated with decreased expression of adrenal catecholamine synthesis genes and an increased heart rate under hypoxic conditions. The highest ranking SNP in the *Epas1* gene resides in exon 14 and encodes for a T755M polymorphism. This SNP is not present in deer mice at sea level, and it rises to a frequency of >0.8 in deer mouse populations at high altitude (21). In the primary sequence, amino acid 755 resides between and is distant from the ODD domain and CTAD. Therefore, it is not intuitively obvious whether the polymorphism has any functional significance. There are no other *Epas1* SNPs under selection that produce a change in the amino acid sequence.

Here we provide evidence that high-altitude deer mouse Hif-2α is a loss of function allele that produces a defect in the interaction of Hif-2α with Cbp. This provides a framework for understanding high-altitude adaptation in deer mice and shows a naturally occurring mutation in a mammalian *Epas1* gene that affects the function of the CTAD of Hif-2α. More broadly, it provides important information for understanding convergent evolution at the *Epas1* locus in mammalian high-altitude species.

## Results and discussion

**Figure 1:**
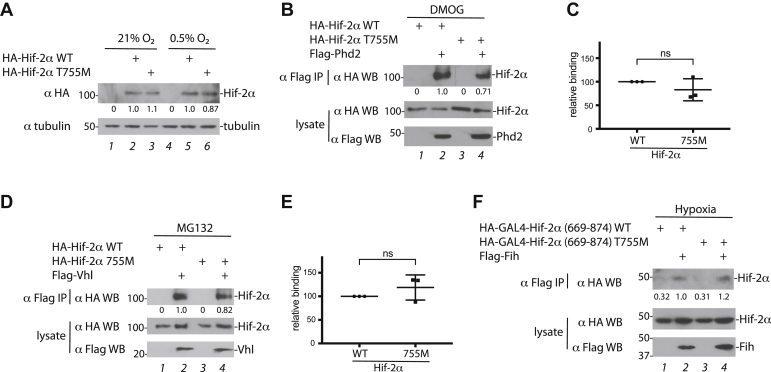
**The high-altitude deer mouse Hif-2α T755M mutation maintains interaction with Phd2, Vhl, and Fih.***A*, HEK293FT cells were transfected and then maintained under normoxia (21% O_2_) or subjected to 18 h of hypoxia (0.5% O_2_). Equal protein extract amounts were examined by Western blotting. Positions of molecular weight markers (in kDa) are indicated here and in subsequent figures. Quantification of band intensities by densitometry is indicated by numbers below bands in this figure and in subsequent figures. *B*–*F*, HEK293FT cells were transfected and then exposed to (*B* and *C*) 1-mM DMOG for 4 h, (*D* and *E*) 100-μM MG-132 for 4 h, or (*F*) 0.5% O_2_ for 18 h. Flag-tagged proteins were immunoprecipitated and Western blotting performed. In panels *C* and *E*, quantitation of three independent experiments of panels *B* and *D*, respectively, is shown. The means ± SD are shown. DMOG, dimethyloxalylglycine; Hif-2α, hypoxia-inducible factor-2α; ns, not significant by student’s *t* test.

To test whether the T755M mutation affects protein stability, we transfected HEK293FT cells with constructs for WT and T755M deer mouse Hif-2α, exposed some cells to hypoxia (0.5% O_2_), and then examined protein levels by Western blotting. We did not observe any significant difference in protein levels between WT and T755M deer mouse Hif-2α under either normoxia or hypoxia (Fig. 1*A*, lanes 2 and 3, and lanes 5 and 6).

The ODD domain of Hif-2α interacts with two proteins—Phd2 when not hydroxylated, and Vhl when hydroxylated. We examined these interactions. We were not able to detect binding between Hif-2α and Phd2 under hypoxia (data not shown). As an alternative, we used dimethyloxalylglycine, an active site inhibitor of 2-oxoglutarate dependent enzymes (which includes Phd2), that allows for the formation of enzyme:substrate complexes (22). We transfected cells with WT or T755M deer mouse Hif-2α constructs along with one for Phd2, exposed cells to dimethyloxalylglycine, and examined Phd2 immunoprecipitates for Hif-2α. We do not see any appreciable differences in the interaction of Phd2 with WT and T755M deer mouse Hif-2α (Fig. 1, *B* and *C*). To examine the binding of Vhl with hydroxylated Hif-2α, we transfected cells with the Hif-2α constructs along with one encoding for deer mouse Vhl. We treated cells with the proteasome inhibitor MG-132 to stabilize the prolyl hydroxylated from of Hif-2α. We observe no significant differences in the amount of WT and T755M Hif-2α recovered in Vhl immunoprecipitates (Fig. 1, *D* and *E*).

The CTAD (residues 834–874) of Hif-2α can bind Fih. We fused WT or T755M deer mouse Hif-2α (669–874), which contains the CTAD, to the DNA-binding domain of the GAL4 protein. We transfected cells with these constructs along with one for deer mouse Fih and exposed cells to hypoxia (0.5% O_2_) to stabilize the nonhydroxylated form of Hif-2α. Hif-2α (669–874) is detectable in Fih immunoprecipitates, and we do not see a difference in the interaction with Fih between WT and T755M deer mouse Hif-2α (669–874) (Fig. 1*F*, lanes 2 and 4).

**Figure 2:**
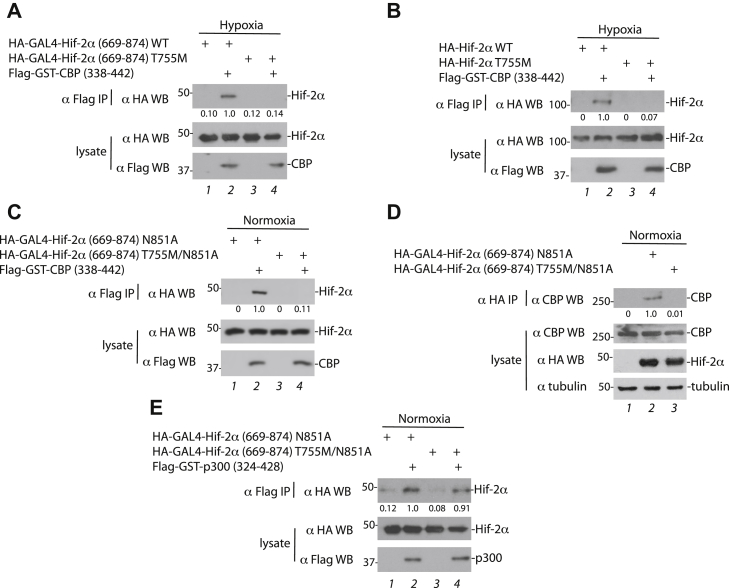
**The high-altitude deer mouse Hif-2α T755M mutation impairs its interaction with Cbp.***A*–*C* and *E*, HEK293FT or (*D*) N2a cells were transfected and then exposed (*A* and *B*) to 0.5% O_2_ for 18 h, or (*C*–*E*) normoxia. *A*–*C* and *E*, flag-tagged or (*D*) hemagglutinin (HA)-tagged proteins were immunoprecipitated and Western blotting performed. CBP, CREB-binding protein; Hif-2α, hypoxia-inducible factor-2α.

In its nonhydroxylated form, the CTAD can interact with Cbp through the zinc finger–containing CH1 (also known as TAZ1) domain of the latter (23). We transfected cells with the Hif-2α (669–874) constructs along with one for the CH1 domain of deer mouse Cbp (residues 338–442, fused to GST). We exposed the cells to hypoxia (0.5% O_2_) to block hydroxylation and examined Cbp immunoprecipitates for Hif-2α. In contrast to the interactions with Phd2, Vhl, and Fih, the T755M mutation in Hif-2α dramatically impairs the interaction with Cbp (Fig. 2*A*). We observed the same result with full-length WT and T755M Hif-2α (Fig. 2*B*). Longer exposures of these Western blots reveal a weak Hif-2α band in the Cbp immunoprecipitates. Therefore, the mutation is hypomorphic, that is, it produces a partial but not complete loss of function.

Previous studies have shown that an Asn > Ala substitution at the site of Fih-catalyzed hydroxylation (Asn-851 in deer mouse Hif-2α) allows constitutive Cbp binding and CTAD activation under normoxia (12). Coimmunoprecipitation studies using Hif-2α (669–874) N851A show that this substitution (N851A) in Hif-2α allows CTAD:Cbp binding under normoxia in a manner sensitive to the T755M mutation (Fig. 2*C*, lanes 2 and 4). We assessed the interaction of these two Hif-2α (669–874) proteins with endogenous Cbp in a murine cellular environment by transfecting mouse N2a cells with the Hif-2α (669–874) constructs and then immunoprecipitating the Hif-2α. The T755M mutation impairs the interaction with endogenous Cbp in this experimental setting (Fig. 2*D*, lanes 2 and 3).

The protein p300 is a paralogue of Cbp with a homologous zinc finger that interacts with the CTAD (24). We examined the interaction of Hif-2α (669–874) N851A with the CH1 zinc finger domain of deer mouse p300 (residues 324–428). In contrast to Cbp, binding is maintained with the T755M mutation (Fig. 2*E*, lanes 2 and 4). Although we cannot exclude the possibility that there might be subtle effects on the binding of the T755M mutant to Phd2, Vhl, Fih, or p300, the results indicate that the T755M mutation in deer mouse Hif-2α primarily impairs interaction with Cbp.

**Figure 3:**
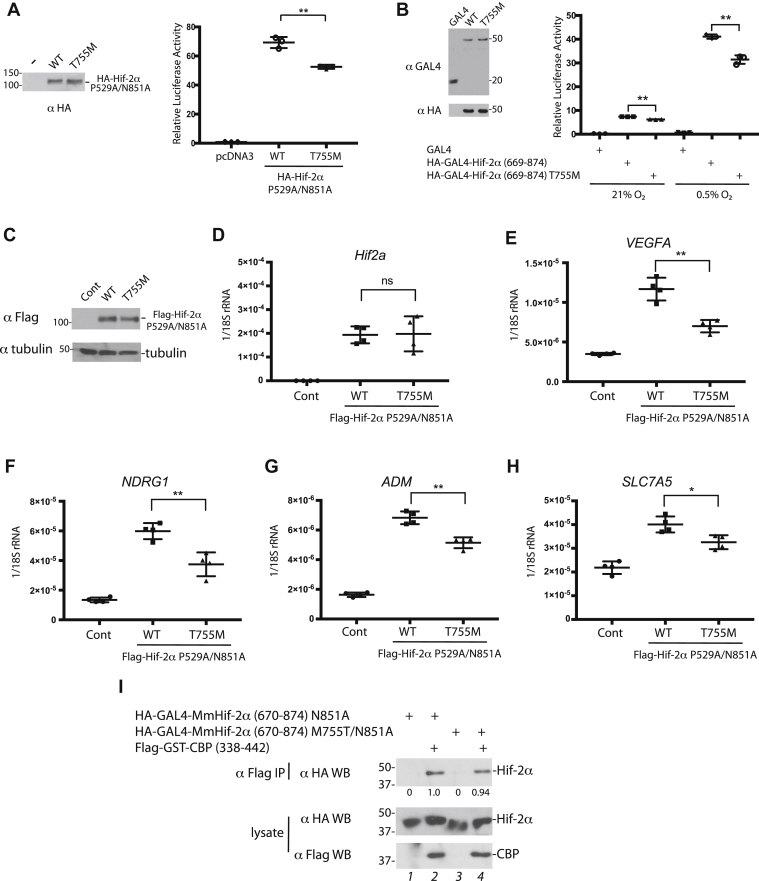
**The high-altitude deer mouse Hif-2α T755M mutation impairs transcriptional activity.***A*, *Left*, HEK293FT cells were transfected, and equal protein extract amounts examined by Western blotting. *Right*, cells were transfected 10 ng of (eHRE)_3_-Luc, 10 ng of RL-TK, and 30 ng of plasmids expressing the indicated proteins. WT indicates that the amino acid at residue 755 is Thr. Twenty hours after transfection, luciferase activities were measured. *B*, *Left*, HEK293FT cells were transfected with plasmids expressing GAL4 or either WT or T755M HA-GAL4-Hif-2α (669–874). Equal protein extract amounts were examined by Western blotting. *Right*, cells were transfected 20 ng of (GAL4)_5_-E1b-Luc, 20 ng of RL-TK, and 30 ng of plasmids expressing the indicated proteins. Four hr after transfection, cells were exposed to 0.5% O_2_ or maintained under normoxia for an additional 18 h and luciferase activities measured. *C*, Flp-In TRex 293 cells stably transfected with the indicated constructs or parental Flp-In TRex 293 cells (Cont) were induced with tetracycline. Equal protein extract amounts were examined by Western blotting. *D*–*H*, RNA was harvested from these cells, reverse transcribed, and then real-time PCR performed to measure the transcript levels from (*D*) the deer mouse *Hif2a* transgene, and the genes for (*E*) vascular endothelial growth factor A (*VEGFA*), (*F*) N-myc downstream regulated gene 1 (*NDRG1*), (*G*) adrenomedullin (*ADM*), and (*H*) solute carrier family 7 member 5 (*SLC7A5*). *I*, HEK293FT cells were transfected, flag-tagged proteins were immunoprecipitated, and Western blotting was performed. In panels *A*, *B* and *D*–*H*, ∗∗*p* < 0.01, ∗*p* < 0.05, and ns, not significant by Student's *t* test. The means ± SD are shown. Hif-2α, hypoxia-inducible factor-2α.

To assess transcriptional activity, we transfected 293FT cells with constructs for full-length Hif-2α–bearing mutations at the prolyl (P529A) and asparaginyl (N851A) sites that lead to constitutive activity under normoxia (to allow assessment of Hif-2α activity in the absence of endogenous HIF), along with a luciferase reporter gene driven by three copies of hypoxia response element from the *ERYTHROPOIETIN* gene (25). In the context of the P529A/N851A substitutions, the T755M mutant of deer mouse Hif-2α has lower transcriptional activity than WT (Fig. 3*A*). We examined the transcriptional activity of the GAL4–Hif-2α (669–874) fusion protein (without the N581A mutation) under normoxia and hypoxia with the use of a luciferase reporter gene driven by GAL4 binding sites. Hypoxia induces higher reporter gene activity from the WT Hif-2α (669–874) fusion protein, and the T755M mutation confers lower activity under either normoxia or hypoxia (Fig. 3*B*). The activity of Hif-2α is only partially reduced in these reporter gene assays, consistent with the partial (as opposed to complete) loss of interaction with Cbp and the preservation of interaction with p300.

We assessed the effect of the T755M mutation on endogenous Hif-2α gene targets by generating HEK293 cells stably expressing WT and T755M deer mouse Hif-2α from an isogenic locus (using the Flp-In TRex system) (Fig. 3, *C* and *D*). These two constructs were in the P529A/N851A background to allow assessment under normoxia and avoid interference from endogenous HIF. Deer mouse Hif-2α induces transcriptional activation of the HIF-2α target genes *VEGFA*, *NDRG1*, *ADM*, and *SLC7A5* (Fig. 3, *E*–*H*). Importantly, the T755M mutation diminishes activation of these genes.

The deer mouse mutation changes residue 755 to an amino acid, methionine, that is the native amino acid at residue 755 in house mouse (*Mus musculus*) Hif-2α (MmHif-2α). We examined the effect of the converse substitution, M755T, on the interaction between house mouse Hif-2α with house mouse Cbp (338–442), the sequence of which is identical to deer mouse Cbp (338–442). In contrast to deer mouse Hif-2α, we find that both WT (M755) and M755T house mouse Hif-2α (670–874) interact with Cbp (Fig. 3*I*, lanes 2 and 4). Therefore, the interchange between methionine and threonine at this position in house mouse Hif-2α lacks the same functional effect that is observed in deer mouse Hif-2α.

**Figure 4:**
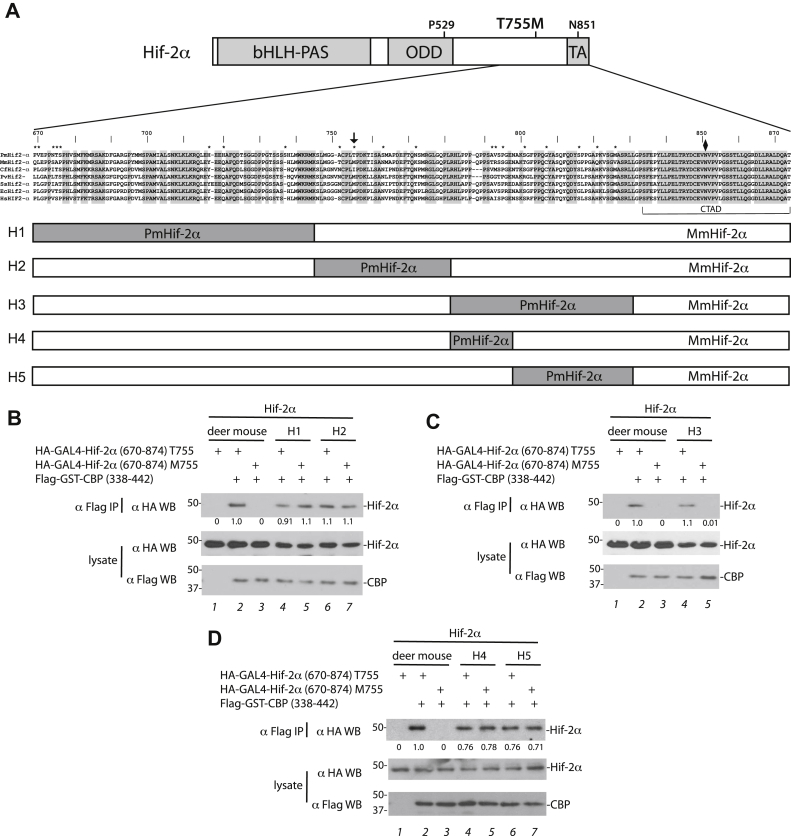
**The deer mouse Hif-2α background allows the T755M substitution to produce a functional defect.***A*, the diagram of deer mouse (*Peromyscus maniculatus bairdii*, XP_015860532.1) Hif-2α. Sequence of residues 669 to 874 shown, along with corresponding sequences of Hif-2α from house mouse (*Mus musculus*, NP_034267.3), dog (*Canis familiaris*, XP_005626137.1), harbor seal (*Phoca vitulina*, XP_032266944.1), pig (*Sus scrofa*, NP_001090889.1), horse (*Equus caballus*, XP_005600062.1), and human (*Homo sapiens*, NP_001421.2). Alignment produced using the Clustal W method (Lasergene MegAlign v15.3.0). Shading indicates residues conserved in all species. *Arrow* = Thr-755 in deer mouse Hif-2α. *Diamond* = Asn-851. *Asterisks* indicate residues that are dissimilar between deer mouse and house mouse Hif-2α. The numbers above sequences refer to deer mouse Hif-2α residues. In hybrid constructs, deer mouse and house mouse Hif-2α sequences are *shaded* and *white*, respectively. *B*–*D*, HEK293FT cells were transfected, flag-tagged proteins were immunoprecipitated, and Western blotting was performed. All Hif-2α constructs have an N851A substitution. Hif-2α, hypoxia-inducible factor-2α.

Besides amino acid 755, deer mouse Hif-2α (669–874) differs from house mouse Hif-2α (670–874) at 19 additional residues (Fig. 4*A*). This suggests that the loss of function effect of the T755M mutation in Hif-2α is only seen in the context of amino acid substitutions that are present in deer mouse Hif-2α (669–874). We prepared hybrid constructs in which specific regions of deer mouse Hif-2α (669–874) were inserted into the corresponding sequence of house mouse Hif-2α (residues 670–874) to determine which could confer sensitivity to the T755M mutation (Fig. 4*A*). We prepared five hybrid constructs, designated H1 to H5, that span the entire region of dissimilarity between deer mouse and house mouse Hif-2α and prepared T755 and M755 versions of each. The first two of these, H1 and H2, behave similarly to house mouse Hif-2α (670–874), that is, the T755M mutation preserves Cbp binding (Fig. 4*B*). In contrast, with H3, the T755M mutation impairs binding (Fig. 4*C*, lanes 4 and 5). In additional hybrids in which the deer mouse region of H3 was subdivided into two, producing H4 and H5 hybrids, we found that the T755M mutation maintains binding to Cbp in both cases, that is, the deleterious effect is lost (Fig. 4*D*). This suggests that multiple deer mouse amino acid substitutions in H3 (which includes T792A, R793V, S795P, T801S, C807G, G816S, Q821P, V826M) are necessary to confer sensitivity of Cbp binding in house mouse Hif-2α to the presence of methionine at residue 755.

**Figure 5:**
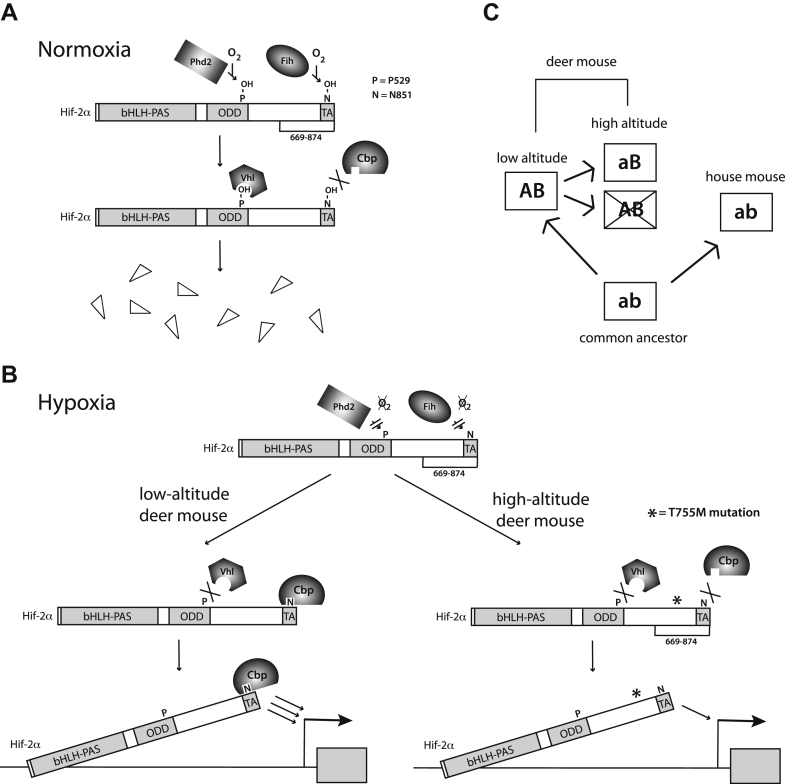
**Model for high-altitude deer mouse Hif-2α.***A*, under normoxia, Phd2-catalyzed prolyl hydroxylation of Hif-2α in the ODD domain promotes Vhl binding and Hif-2α degradation, while Fih-catalyzed asparaginyl hydroxylation of HIF-α in the CTAD (TA) blocks interaction with Cbp. Sites of prolyl hydroxylation (P = P529), asparaginyl hydroxylation (N = N851), and residues 669 to 874 are as indicated. *B*, under hypoxia, these modifications are arrested, resulting in the stabilization of Hif-2α. In the case of low-altitude deer mouse Hif-2α (*left*), the CTAD binds Cbp. In the case of high-altitude deer mouse Hif-2α (*right*), the T755M substitution (indicated by an *asterisk*) impairs interaction between the CTAD and Cbp. The number of arrows between the CTAD and the gene indicate strength of transcriptional activation. *C*, proposed emergence of the T755M substitution in high-altitude deer mice. *Boxes* indicate Hif-2α. *Letters within boxes* indicate amino acids as follows. a = Met-755, A = Thr-755. b = house mouse Hif-2α background, B = deer mouse Hif-2α background (see Fig. 4*A* for specific amino acid substitutions). The “aB” allele arises from an “AB” allele. The *cross* indicates a maladaptive allele. The diagram is adapted from Figure 1 of (27).

Under normoxia, prolyl and asparaginyl hydroxylation inhibit Hif-2α (Fig. 5*A*), whereas under hypoxia, this inhibition is relieved (Fig. 5*B*, *left*). We find that the North American high-altitude deer mouse T755M mutation in Hif-2α produces a loss of function effect *via* impaired binding to Cbp (Fig. 5*B*, *right*). *In vivo*, the corresponding SNP in the *Epas1* gene is associated with decreased levels of catecholamine biosynthetic gene expression in the adrenal gland (21). Because previous observations indicated that HIF-2α is required for catecholamine synthesis in sympathoadrenal cells (26), our results could provide a mechanistic explanation for this. The resulting vasodilation might lead to compensatory increased heart rate, which has been observed (21).

Residue 755 is in a region of deer mouse Hif-2α that resides between the ODD domain and CTAD (Fig. 4*A*) and is not as highly conserved as either the ODD domain or CTAD. We find that the differences in the behavior of Thr and Met at this position are context dependent and seen only in deer mouse, but not house mouse, Hif-2α. This depends on a region in deer mouse Hif-2α (residues 792–826) that includes multiple amino acid substitutions. This may represent an example of intramolecular epistasis (27), somewhat analogous to the intramolecular epistasis that has been observed in high-altitude deer mouse hemoglobin (28). We propose the following. Deer mouse and house mouse Hif-2α arose from a common ancestor (Fig. 5*C*). The ancestral M755 is indicated by “a”, whereas the other ancestral amino acids are indicated by “b”. Low-altitude deer mouse Hif-2α acquired amino acid substitutions that are indicated by “A” for M755T and “B” for the 19 other amino acid changes (Fig. 4*A*). The “AB” haplotype in deer mice is neutral under low-altitude conditions but is maladaptive at a high altitude. The “B” substitutions, however, provide a genetic background in which a reversion of “A” to the ancestral “a” allows the acquisition of a functional adaptive change in Hif-2α at high altitude (*i.e.*, the “aB” haplotype). In a “b” background, interchange between “a” and “A” is otherwise neutral.

No three-dimensional structure is available for the region in Hif-2α in which Thr-755 resides, and it is conceivable that it might be a disordered region. If so, Thr-755 of Hif-2α may bear similarity to residue 127 in human PHD2, which is in a poorly conserved, possibly disordered, region of PHD2 located between a zinc finger and the catalytic domain of PHD2. Tibetans harbor a C127S substitution in PHD2 that, in the context of the WT protein, is without an appreciable effect *in vitro*. However, in the context of a D4E mutation that is adjacent to the PHD2 zinc finger, the C127S substitution results in markedly impaired interaction of PHD2 with the HSP90 cochaperone p23, leading to loss of function (29, 30). We propose that the high-altitude deer mouse T755M Hif-2α mutation and the human Tibetan C127S PHD2 substitution represent two independent adaptive amino acid changes in the HIF pathway that occur in potentially disordered regions of the respective proteins, produce loss of function in the context of other selective amino acid changes, and promote adaptation to the chronic hypoxia of high altitude.

## Experimental procedures

Experimental procedures are available in Supporting information. Each experiment was performed at least two times. For some experiments, data were analyzed by two-tailed student’s *t* test (GraphPad Prism 7).

## Data availability

All data relevant to these studies are contained within the article.

## Supporting information

This article contains supporting information (25, 31, 32, 33, 34, 35, 36).

## Conflict of interest

The authors declare that they have no conflicts of interest with the contents of this article.
