# Supplementary material for: High-altitude deer mouse hypoxia-inducible factor-2α shows defective interaction with CREB-binding protein
Source: J Biol Chem. 2021 Feb 25;296:100461. doi: 10.1016/j.jbc.2021.100461 (PMC8024697; doi:10.1016/j.jbc.2021.100461)
Supplement: Supporting Information [file mmc1.pdf]

Supporting Information

High-altitude deer mouse Hypoxia inducible factor-2α shows defective interaction with CREB-binding protein

Daisheng Song[1], Abigail W. Bigham[2], and Frank S. Lee[1]*

From the [1]Department of Pathology and Laboratory Medicine, Perelman School of Medicine, University of Pennsylvania, Philadelphia, Pennsylvania 19104, and [2]Department of Anthropology, University of California, Los Angeles, CA 90095

Experimental procedures

Plasmids.

Unless otherwise noted, the plasmids described encode for proteins from deer mouse (*Peromyscus maniculatus bairdii*). pcDNA3.1-HA-Hif-2α, pcDNA3.1- HA-Hif-2α T755M/N851A, pcDNA3.1- HA-Hif-2α P529A/N851A, pcDNA3.1- HA-Hif-2α P529A/T755M/N851A, pcDNA3.1-Flag-Fih, and pcDNA3.1-Flag-Vhl were obtained from Genscript. pcDNA3.1-HA-Hif-2α T755M was constructed by subcloning a 0.33 kb EcoR I/Xho I fragment of pcDNA3.1- HA-Hif-2α into the EcoR I/Xho I site of pcDNA3.1- HA-Hif-2α T755M/N851A. pcDNA3.1-Flag-Phd2 (which encodes for Phd2 from a congeneric species *Peromyscus leucopus*; the Phd2 sequence from *Peromyscus maniculatus bairdii* was not available) was obtained from Genscript. pcDNA5/FRT/TO-3xFlag-Hif-2α P529A/N851A was generated by subcloning the 2.6 kb BamH I/Xho I fragment of pcDNA3.1-HA-Hif-2α P529A/N851A into the BamH I/Xho I site of pcDNA5/FRT/TO. pcDNA5/FRT/TO-3xFlag-Hif-2α P529A/T755M/N851A was generated by subcloning the 2.6 kb BamH I/Xho I fragment of pcDNA3.1-HA-Hif-2α P529A/T755M/N851A into the BamH I/Xho I site of pcDNA5/FRT/TO.

pcDNA3-GAL4-3xHA-Hif2 (669-874), pcDNA3-GAL4-3xHA-Hif2 (669-874) T755M, pcDNA3-GAL4-3xHA-Hif2 (669-874) N851A, and pcDNA3-GAL4-3xHA-Hif2 (669-874) T755M/N851A were prepared as follows. First, the following DNA fragments were synthesized by IDT. CTAD WT: 5'
GTACGGATCCAACCGGTGGAGCCGCCCAACACCTCACCTCACGTCTCCATGTTCAAGATGAG
GTCTGCCAAGGACTTCGGGGCTCGCGGCCCGTACATGATGAGCCCCGCCATGATAGCCCTCT
CCAACAAGCTCAAGCTGAAGCGCCAGCTGGAACACGAGGAGCACGCCTTCCAAGACACGAG
CGGGGGGGACCCTCCGGGCACCAGCAGCAGTTCACACTTGATGTGGAAACGAATGAAGAGC
CTCATGGGTGGGGCCTGTCCTTTGACGCCCGACAAGACCATCAGTGCAAGCATGGCCCCTGA
TGAATTCACCCAAAACTCCATGAGAGGCCTGGGCCAGCCACTGAGACACCTGCCACCCCCGC
AGCCACCATCTGCCGTGAGCCCAGGGGAGAATGCCAAGAGTGGGTTCCCGCCACAGGGCTA
TGCCTCCCAGTTCCAGGACTACAGTCCTCCAGGAGCCCCGAAGGTGTCAGGCATGGCTAGCC
GACTGCTGGGGCCATCGTTCGAGCCTTACCTGTTGCCGGAACTGACCCGATATGACTGTGAG
GTGAATGTCCCTGTGCCGGGAAGTTCCACACTCCTGCAAGGGAGAGACCTTCTCAGAGCTCT
GGACCAGGCCACCTCTAGATACCCATACGATGTTCCAGATTACGCTGGCTATCCCTATGACG
TACCGGACTATGCCGGATCTTATCCTTACGACGTACCTGATTACGCATAACTCGAGCGGCCG
CGGGCCCTA 3'. CTAD T755M: 5'
GTACGGATCCAACCGGTGGAGCCGCCCAACACCTCACCTCACGTCTCCATGTTCAAGATGAG
GTCTGCCAAGGACTTCGGGGCTCGCGGCCCGTACATGATGAGCCCCGCCATGATAGCCCTCT
CCAACAAGCTCAAGCTGAAGCGCCAGCTGGAACACGAGGAGCACGCCTTCCAAGACACGAG
CGGGGGGGACCCTCCGGGCACCAGCAGCAGTTCACACTTGATGTGGAAACGAATGAAGAGC
CTCATGGGTGGGGCCTGTCCTTTGATGCCCGACAAGACGATATCTGCAAGCATGGCCCCTGA
TGAATTCACCCAAAACTCCATGAGAGGCCTGGGCCAGCCACTGAGACACCTGCCACCCCCGC
AGCCACCATCTGCCGTGAGCCCAGGGGAGAATGCCAAGAGTGGGTTCCCGCCACAGGGCTA
TGCCTCCCAGTTCCAGGACTACAGTCCTCCAGGAGCCCCGAAGGTGTCAGGCATGGCTAGCC
GACTGCTGGGGCCATCGTTCGAGCCTTACCTGTTGCCGGAACTGACCCGATATGACTGTGAG
GTGAATGTCCCTGTGCCGGGAAGTTCCACACTCCTGCAAGGGAGAGACCTTCTCAGAGCTCT
GGACCAGGCCACCTCTAGATACCCATACGATGTTCCAGATTACGCTGGCTATCCCTATGACG
TACCGGACTATGCCGGATCTTATCCTTACGACGTACCTGATTACGCATAACTCGAGCGGCCG
CGGGCCCTA 3'. CTAD N851A: 5'
GTACGGATCCAACCGGTGGAGCCGCCCAACACCTCACCTCACGTCTCCATGTTCAAGATGAG
GTCTGCCAAGGACTTCGGGGCTCGCGGCCCGTACATGATGAGCCCCGCCATGATAGCCCTCT
CCAACAAGCTCAAGCTGAAGCGCCAGCTGGAACACGAGGAGCACGCCTTCCAAGACACGAG
CGGGGGGGACCCTCCGGGCACCAGCAGCAGTTCACACTTGATGTGGAAACGAATGAAGAGC

CTCATGGGTGGGGCCTGTCCTTTGACGCCCGACAAGACCATCAGTGCAAGCATGGCCCCTGA
TGAATTCACCCAAAACTCCATGAGAGGCCTGGGCCAGCCACTGAGACACCTGCCACCCCCGC
AGCCACCATCTGCCGTGAGCCCAGGGGAGAATGCCAAGAGTGGGTTCCCGCCACAGGGCTA
TGCCTCCCAGTTCCAGGACTACAGTCCTCCAGGAGCCCCGAAGGTGTCAGGCATGGCTAGCC
GACTGCTGGGGCCATCGTTCGAGCCTTACCTGTTGCCGGAACTGACCCGATATGACTGTGAG
GTGGCTGTCCCTGTGCCCGGGAGTTCCACACTCCTGCAAGGGAGAGACCTTCTCAGAGCTCT
GGACCAGGCCACCTCTAGATACCCATACGATGTTCCAGATTACGCTGGCTATCCCTATGACG
TACCGGACTATGCCGGATCTTATCCTTACGACGTACCTGATTACGCATAACTCGAGCGGCCG
CGGGCCCTA 3’. CTAD T755M/N851A: 5’
GTACGGATCCAACCGGTGGAGCCGCCCAACACCTCACCTCACGTCTCCATGTTCAAGATGAG
GTCTGCCAAGGACTTCGGGGCTCGCGGCCCGTACATGATGAGCCCCGCCATGATAGCCCTCT
CCAACAAGCTCAAGCTGAAGCGCCAGCTGGAACACGAGGAGCACGCCTTCCAAGACACGAG
CGGGGGGGACCCTCCGGGCACCAGCAGCAGTTCACACTTGATGTGGAAACGAATGAAGAGC
CTCATGGGTGGGGCCTGTCCTTTGATGCCCGACAAGACGATATCTGCAAGCATGGCCCCTGA
TGAATTCACCCAAAACTCCATGAGAGGCCTGGGCCAGCCACTGAGACACCTGCCACCCCCGC
AGCCACCATCTGCCGTGAGCCCAGGGGAGAATGCCAAGAGTGGGTTCCCGCCACAGGGCTA
TGCCTCCCAGTTCCAGGACTACAGTCCTCCAGGAGCCCCGAAGGTGTCAGGCATGGCTAGCC
GACTGCTGGGGCCATCGTTCGAGCCTTACCTGTTGCCGGAACTGACCCGATATGACTGTGAG
GTGGCTGTCCCTGTGCCCGGGAGTTCCACACTCCTGCAAGGGAGAGACCTTCTCAGAGCTCT
GGACCAGGCCACCTCTAGATACCCATACGATGTTCCAGATTACGCTGGCTATCCCTATGACG
TACCGGACTATGCCGGATCTTATCCTTACGACGTACCTGATTACGCATAACTCGAGCGGCCG
CGGGCCCTA 3’. Then, these DNA fragments were digested with BamH I/Not I and subcloned into the BamH I/Not I site of pcDNA3-GAL4.

pcDNA3-GAL4-3xHA-MmHif2 (670-874) N851A and pcDNA3-GAL4-3xHA-MmHif2 (670-874) M755T/N851A were constructed in an analogous manner (Mm = *Mus musculus*). The sequences of the DNA fragments were as follows: MmCTAD N851A: 5’
GTACGGATCCAACAGTTGGAACCTCCGAGCGCCCCGCCTCATGTCTCCATGTTCAAGATGAG
GTCTGCAAAGGACTTCGGGGCCCGAGGTCCATACATGATGAGCCCAGCCATGATCGCCCTGT
CCAACAAGCTGAAGCTAAAGCGGCAGCTGGAGTATGAGGAGCAAGCCTTCCAAGACACAAG
CGGGGGGGACCCTCCAGGCACCAGCAGTTCACACTTGATGTGGAAACGTATGAAGAGCCTC
ATGGGCGGGACCTGTCCTTTGATGCCTGACAAGACCATCAGTGCGAACATGGCCCCCGATGA
ATTCACCCAAAAATCTATGAGAGGCCTGGGCCAGCCACTGAGACACCTGCCACCTCCCCAGC
CACCATCTACCAGGAGCTCAGGGGAGAACGCCAAGACTGGGTTCCCGCCACAGTGCTATGC
CTCCCAGTTCCAGGACTACGGTCCTCCAGGAGCTCAAAAGGTGTCAGGCGTGGCCAGTCGAC
TGCTGGGGCCATCGTTCGAGCCTTACCTGTTGCCGGAACTGACCAGATATGACTGTGAGGTG
GCCGTGCCCGTGCCCGGGAGCTCCACACTCCTGCAGGGGAGAGACCTTCTCAGAGCTCTGGA
CCAGGCCACCTCTAGATACCCATACGATGTTCCAGATTACGCTGGCTATCCCTATGACGTAC
CGGACTATGCCGGATCTTATCCTTACGACGTACCTGATTACGCATAACTCGAGCGGCCGCGG
GCCCTA 3’. MmCTAD M755T/N851A: 5’
GTACGGATCCAACAGTTGGAACCTCCGAGCGCCCCGCCTCATGTCTCCATGTTCAAGATGAG
GTCTGCAAAGGACTTCGGGGCCCGAGGTCCATACATGATGAGCCCAGCCATGATCGCCCTGT
CCAACAAGCTGAAGCTAAAGCGGCAGCTGGAGTATGAGGAGCAAGCCTTCCAAGACACAAG
CGGGGGGGACCCTCCAGGCACCAGCAGTTCACACTTGATGTGGAAACGTATGAAGAGCCTC
ATGGGCGGGACCTGTCCTTTGACGCCTGACAAGACGATATCTGCGAACATGGCCCCCGATGA
ATTCACCCAAAAATCTATGAGAGGCCTGGGCCAGCCACTGAGACACCTGCCACCTCCCCAGC
CACCATCTACCAGGAGCTCAGGGGAGAACGCCAAGACTGGGTTCCCGCCACAGTGCTATGC
CTCCCAGTTCCAGGACTACGGTCCTCCAGGAGCTCAAAAGGTGTCAGGCGTGGCCAGTCGAC
TGCTGGGGCCATCGTTCGAGCCTTACCTGTTGCCGGAACTGACCAGATATGACTGTGAGGTG
GCCGTGCCCGTGCCCGGGAGCTCCACACTCCTGCAGGGGAGAGACCTTCTCAGAGCTCTGGA
CCAGGCCACCTCTAGATACCCATACGATGTTCCAGATTACGCTGGCTATCCCTATGACGTAC
CGGACTATGCCGGATCTTATCCTTACGACGTACCTGATTACGCATAACTCGAGCGGCCGCGG

GCCCTA 3'. These DNA fragments were digested with BamH I/Not I and subcloned into the BamH I/Not I site of pcDNA3-GAL4.

pcDNA3-GAL4-3xHA-Hif-2α (670-874) H1 T755/N851A, pcDNA3-GAL4-3xHA-Hif-2α (670-874) H1 M755/N851A, pcDNA3-GAL4-3xHA-Hif-2α (670-874) H2 T755/N851A, pcDNA3-GAL4-3xHA-Hif-2α (670-874) H2 M755/N851A, pcDNA3-GAL4-3xHA-Hif-2α (670-874) H3 T755/N851A, and pcDNA3-GAL4-3xHA-Hif-2α (670-874) H3 M755/N851A were constructed as follows. First, the following DNA fragments were synthesized by IDT. CTAD H1 T755: 5'
GTACGGATCCAACCGGTGGAGCCGCCCAACACCTCACCTCACGTCTCCATGTTCAAGATGAG
GTCTGCCAAGGACTTCGGGGCTCGCGGCCCGTACATGATGAGCCCCGCCATGATAGCCCTCT
CCAACAAGCTCAAGCTGAAGCGCCAGCTGGAACACGAGGAGCACGCCTTCCAAGACACGAG
CGGGGGGGACCCTCCAGGTACCAGCAGCAGTTCACACTTGATGTGGAAACGTATGAAGAGC
CTCATGGGCGGGACCTGTCCTTTGACGCCTGACAAGACCATCAGTGCGAACATGGCCCCCGA
TGAATTCACCCAAAAATCTATGAGAGGCCTGGGCCAGCCCTTAAGACACCTGCCACCTCCCC
AGCCACCATCTACCAGGAGCTCAGGGGAGAACGCCAAGACTGGGTTCCCGCCACAGTGCTA
TGCCTCCCAGTTCCAGGACTATGGTCCTCCAGGAGCTCAAAAGGTGTCAGGCGTGGCTAGCC
GACTG 3'. CTAD H1 M755: 5'
GTACGGATCCAACCGGTGGAGCCGCCCAACACCTCACCTCACGTCTCCATGTTCAAGATGAG
GTCTGCCAAGGACTTCGGGGCTCGCGGCCCGTACATGATGAGCCCCGCCATGATAGCCCTCT
CCAACAAGCTCAAGCTGAAGCGCCAGCTGGAACACGAGGAGCACGCCTTCCAAGACACGAG
CGGGGGGGACCCTCCAGGTACCAGCAGCAGTTCACACTTGATGTGGAAACGTATGAAGAGC
CTCATGGGCGGGACCTGTCCTTTGATGCCTGACAAGACGATATCTGCGAACATGGCCCCCGA
TGAATTCACCCAAAAATCTATGAGAGGCCTGGGCCAGCCCTTAAGACACCTGCCACCTCCCC
AGCCACCATCTACCAGGAGCTCAGGGGAGAACGCCAAGACTGGGTTCCCGCCACAGTGCTA
TGCCTCCCAGTTCCAGGACTATGGTCCTCCAGGAGCTCAAAAGGTGTCAGGCGTGGCTAGCC
GACTG 3'. CTAD H2 T755: 5'
GTACGGATCCAACAGTTGGAACCTCCGAGCGCCCCGCCTCATGTCTCCATGTTCAAGATGAG
GTCTGCAAAGGACTTCGGGGCCCGAGGTCCATACATGATGAGCCCAGCCATGATCGCCCTGT
CCAACAAGCTGAAGCTAAAGCGGCAGCTGGAGTATGAGGAGCAAGCCTTCCAAGACACAAG
CGGGGGGGACCCTCCGGGTACCAGCAGTTCACACTTGATGTGGAAACGAATGAAGAGCCTC
ATGGGTGGGGCCTGTCCTTTGACGCCCGACAAGACCATCAGTGCAAGCATGGCCCCTGATGA
ATTCACCCAAAACTCCATGAGAGGCCTGGGCCAGCCCTTAAGACACCTGCCACCCCCGCAGC
CACCATCTACCAGGAGCTCAGGGGAGAACGCCAAGACTGGGTTCCCGCCACAGTGCTATGC
CTCCCAGTTCCAGGACTATGGTCCTCCAGGAGCTCAAAAGGTGTCAGGCGTGGCTAGCCGAC
TG 3'. CTAD H2 M755: 5'
GTACGGATCCAACAGTTGGAACCTCCGAGCGCCCCGCCTCATGTCTCCATGTTCAAGATGAG
GTCTGCAAAGGACTTCGGGGCCCGAGGTCCATACATGATGAGCCCAGCCATGATCGCCCTGT
CCAACAAGCTGAAGCTAAAGCGGCAGCTGGAGTATGAGGAGCAAGCCTTCCAAGACACAAG
CGGGGGGGACCCTCCGGGTACCAGCAGTTCACACTTGATGTGGAAACGAATGAAGAGCCTC
ATGGGTGGGGCCTGTCCTTTGATGCCCGACAAGACGATATCTGCAAGCATGGCCCCTGATGA
ATTCACCCAAAACTCCATGAGAGGCCTGGGCCAGCCCTTAAGACACCTGCCACCCCCGCAGC
CACCATCTACCAGGAGCTCAGGGGAGAACGCCAAGACTGGGTTCCCGCCACAGTGCTATGC
CTCCCAGTTCCAGGACTATGGTCCTCCAGGAGCTCAAAAGGTGTCAGGCGTGGCTAGCCGAC
TG 3'. CTAD H3 T755: 5'
GTACGGATCCAACAGTTGGAACCTCCGAGCGCCCCGCCTCATGTCTCCATGTTCAAGATGAG
GTCTGCAAAGGACTTCGGGGCCCGAGGTCCATACATGATGAGCCCAGCCATGATCGCCCTGT
CCAACAAGCTGAAGCTAAAGCGGCAGCTGGAGTATGAGGAGCAAGCCTTCCAAGACACAAG
CGGGGGGGACCCTCCAGGTACCAGCAGTTCACACTTGATGTGGAAACGTATGAAGAGCCTC
ATGGGCGGGACCTGTCCTTTGACGCCTGACAAGACCATCAGTGCGAACATGGCCCCCGATGA
ATTCACCCAAAAATCTATGAGAGGCCTGGGCCAGCCCTTAAGACACCTGCCACCTCCCCAGC
CACCATCTGCCGTGAGCCCAGGGGAGAATGCCAAGAGTGGGTTCCCGCCACAGGGCTATGC

CTCCCAGTTCCAGGACTACAGTCCTCCAGGAGCCCCGAAGGTGTCAGGCATGGCTAGCCGACTG 3'. CTAD H3 M755: 5' GTACGGATCCAACAGTTGGAACCTCCGAGCGCCCCGCCTCATGTCTCCATGTTCAAGATGAGGTCTGCAAAGGACTTCGGGGCCCGAGGTCCATACATGATGAGCCCAGCCATGATCGCCCTGTCCAACAAGCTGAAGCTAAAGCGGCAGCTGGAGTATGAGGAGCAAGCCTTCCAAGACACAAGCGGGGGGGACCCTCCAGGTACCAGCAGTTCACACTTGATGTGGAAACGTATGAAGAGCCTCATGGGCGGGACCTGTCCTTTGATGCCTGACAAGACGATATCTGCGAACATGGCCCCCGATGAATTCACCCAAAAATCTATGAGAGGCCTGGGCCAGCCCTTAAGACACCTGCCACCTCCCCAGCCACCATCTGCCGTGAGCCCAGGGGAGAATGCCAAGAGTGGGTTCCCGCCACAGGGCTATGCCTCCCAGTTCCAGGACTACAGTCCTCCAGGAGCCCCGAAGGTGTCAGGCATGGCTAGCCGACTG 3'. Then, these DNA fragments were digested with BamH I/Nhe I and subcloned into the BamH I/Nhe I site of pcDNA3-GAL4-3xHA-Hif2 (669-874) N851A.

pcDNA3-GAL4-3xHA-Hif-2α (670-874) H4 T755/N851A, pcDNA3-GAL4-3xHA-Hif-2α (670-874) H4 M755/N851A, pcDNA3-GAL4-3xHA-Hif-2α (670-874) H5 T755/N851A, and pcDNA3-GAL4-3xHA-Hif-2α (670-874) H5 M755/N851A were constructed as follows. First, the following DNA fragments were synthesized by IDT. CTAD H4: 5' ATGAGAGGTTTAGGACAACCCTTAAGACACCTGCCACCTCCCCAGCCACCATCCGCGGTGAGCCCAGGGGAGAACGCCAAGACTGGGTTCCCGCCACAGTGCTATGCCTCCCAGTTCCAGGACTACGGTCCTCCAGGAGCTCAAAAGGTGTCAGGCGTGGCTAGCAGATTACTA 3'. CTAD H5: 5' ATGAGAGGTTTAGGCCAGCCCTTAAGACACCTGCCACCTCCCCAGCCACCATCTACCAGAAGCTCAGGGGAGAATGCCAAGAGTGGGTTCCCGCCACAGGGCTACGCGTCCCAGTTCCAGGACTACAGTCCTCCAGGAGCCCCGAAGGTGTCAGGCATGGCTAGCAGATTACTA 3'. Then, these DNA fragments were digested with Afl II/Nhe I and subcloned into the Afl II/Nhe I site of pcDNA3-GAL4-3xHA-Hif2 (670-874) H3 T755/N851A or pcDNA3-GAL4-3xHA-Hif2 (670-874) H3 M755/N851A, as appropriate.

pcDNA3-GST-3xFlag-CBP (338-442) and pcDNA3-GST-3xFlag-p300 (324-428), which encode for the CH1 domains of the respective proteins, were prepared as follows. First, the following DNA fragments were synthesized by IDT. CBP CH1: 5' TTAGTACAGATCTGGATCCAGATGCCTAAGAAGAAGCGTAAGGTAGCAATTGCAACAGGCCCCACAGCAGACCCTGAAAAGCGCAAACTGATACAGCAGCAGCTGGTTCTACTGCTTCATGCCCACAAATGTCAGAGACGAGAGCAAGCAAATGGAGAGGTTCGGGCCTGCTCTCTCCCACACTGTCGAACCATGAAAAATGTTTTGAATCACATGACGCATTGTCAGGCTGGGAAAGCCTGCCAAGTTGCCCATTGTGCATCTTCACGACAAATCATCTCTCATTGGAAGAACTGCACACGACATGACTGTCCTGTTTGCCTCCCTTTGAAAAATGCCAGTGACAAGCGAAACCAACAAGACTACAAAGACCATGACGGTGATTATAAAGATCATGACATCGATTACAAGGATGACGATGACAAGTAGCTCGAGTCTAGAGGGCCCGTCGACATGCATG 3'. P300 CH1: 5' TTAGTACAGATCTGGATCCAGATGCCTAAGAAGAAGCGTAAGGTAATGGGTTCTGGAGCACACACAGCTGATCCAGAGAAGCGCAAGCTCATCCAGCAGCAGCTTGTTCTCCTTTTACATGCTCACAAATGCCAGCGCCGGGAGCAGGCTAATGGGGAAGTGAGGCAGTGCAATCTTCCCCACTGTCGAACCATGAAAAATGTTCTAAACCACATGACACACTGCCAGTCAGGCAAGTCCTGTCAAGTGGCACATTGTGCATCTTCTCGACAAATCATTTCACACTGGAAGAATTGTACAAGGCATGATTGTCCTGTGTGTCTTCCTCTCAAAAATGCTGGGGATAAGCGAAATCAACAGGACTACAAAGACCATGACGGTGATTATAAAGATCATGACATCGATTACAAGGATGACGATGACAAGTAGCTCGAGTCTAGAGGGCCCGTCGACAGTCATG 3'. Then, these DNA fragments were digested with BamH I/Xba I and subcloned into the BamH I/Xba I site of pcDNA3-GST.

The sources of $(eHRE)_3$-Luc and GAL4-E1b-Luc (G5E1b-Luc) have been described (25,31).

Cell culture.

HEK293FT, HEK293 Flp-In TREx stable transfectants, and N2a cells were maintained in DMEM/10% FBS/100 IU/ml penicillin/100 μg/ml streptomycin and transfected using Lipofectamine 2000 as described (32). HEK293FT cells were obtained from Invitrogen. The source of N2a cells has been described (31). HEK293 Flp-In TREx stable transfectants were generated using Flp recombinase (pOG44) and either pcDNA5/FRT/TO-3xFlag-Hif-2α P529A/N851A or pcDNA5/FRT/TO-3xFlag-Hif-2α P529A/T755M/N851A according to the instructions of the manufacturer (Invitrogen). A single clone was isolated for either 3xFlag-Hif-2α P529A/N851A or 3xFlag-Hif-2α P529A/T755M/N851A and expanded. Hif-2α expression from these cells was induced by treating the cells with tetracycline for 20 hr.

Chemicals.

DMOG was obtained from Frontier Scientific. MG132 was obtained from Sigma.

Immunoprecipitations.

Cells were lysed in buffer A (50 mM Tris, pH 7.5, 100 mM NaCl, 0.5% Triton X-100) supplemented with mammalian protease inhibitor cocktail (Sigma P8340). The lysates were clarified by centrifugation at 15,800 x g for 10 min at 4 °C. The lysates were then added to 15 μl aliquots of anti-Flag (mAb M2) agarose or anti-HA (mAb HA-7) agarose (Sigma), and rocked for 1 hr at 4 °C. The resins were washed 3 times with buffer A, eluted, and the eluates subjected to SDS-PAGE and western blotting. For immunoprecipitations involving CBP or p300, 10 μM $ZnCl_2$ was included in the buffers.

Western blotting.

The sources of alkaline phosphatase-conjugated antibodies to Flag tag and HA tag have been described (32,33). The antibodies to Cbp (D6C5) and α tubulin (DM1A), as well as alkaline phosphatase-conjugated goat anti-rabbit IgG antibody (#7054), were from Cell Signaling Technology. The antibody to GAL4 (RK5C1) and alkaline phosphatase-conjugated goat anti-mouse IgG antibody were from Santa Cruz Biotechnology. The procedure for western blotting has been described (25). For immunoprecipitation experiments in which cells were exposed to hypoxia, extracts were prepared in a Ruskinn In Vivo 200 Hypoxia workstation. Protein concentrations of extracts was determined using the Pierce Coomassie Plus Assay. Quantitation of western blots was performed by densitometry, and in the experiments examining immunoprecipitation, recovery was normalized to input.

Luciferase reporter gene assays.

HEK293FT cells in 96-well plates were transfected with plasmids using Lipofectamine 2000. Luciferase assays were performed using a Glomax Explorer detection reader and a Dual-Glo Luciferase Assay System. Firefly luciferase activity was normalized to Renilla luciferase activity expressed from a pRL-TK internal transfection control. For reporter gene experiments in which cells were exposed to hypoxia, plates were placed in a Heracell Tri-Gas 150i incubator.

Real Time PCR.

RNA was isolated from stably transfected HEK293 Flp-In TREx cells using Trizol. Real Time PCR for the HIF-2α target genes *VEGFA*, *ADM*, and *NDRG1* (34) was performed using previously described primers and 18S rRNA as the endogenous internal control (35). That for deer mouse *Hif2a* was performed using the following primers: 5'-CTCCATCATGCGGCTAGCTAT-3' and 5'-AGCAGACTGAGGACAGGAGCTT-3'. That for the HIF-2α target *SLC7A5* (36) was performed using the following primers: 5'-GGAACATTGTGCTGGCATTATACA-3' and 5'-CCTCTGTGACGAAATTCAAGTAATTC-3'. Quantification was performed using the $\Delta\Delta C_T$ method.
